# Minimally Invasive vs. Open Surgery for Hallux Valgus: A Meta-Analysis

**DOI:** 10.3389/fsurg.2022.843410

**Published:** 2022-03-21

**Authors:** Linfeng Ji, Ketao Wang, Shenglong Ding, Chengyi Sun, Songmin Sun, Mingzhu Zhang

**Affiliations:** Department of Ankle and Foot Surgery, Beijing Tongren Hospital, Capital Medical University, Beijing, China

**Keywords:** minimally invasive, percutaneous, hallux valgus, bunion, osteotomy

## Abstract

**Purpose:**

In recent years, minimally invasive surgery (MIS) for hallux valgus has emerged and gained popularity. To date, evidence on the benefits of MIS for hallux valgus is still controversial. This updated meta-analysis aimed to comprehensively evaluate the efficiency of MIS vs. open surgery for hallux valgus.

**Methods:**

A systematic literature search of PubMed, Embase, and the Cochrane Library was performed. Two independent reviewers conducted data extraction and analyzed data with R software. Data were presented with risk ratio (RR) and standardized mean difference (SMD) along with 95% confidence interval (CI).

**Results:**

A total of 22 studies in which there were 790 ft treated with the MIS procedure and 838 ft treated with an open procedure were included. The correction of sesamoid position was better in the MIS group. The post-operative distal metatarsal articular angle (DMAA) of the MIS group was lower. There was less pain at the early phase in the MIS group. The MIS group had a shorter surgery time and shorter hospitalization time compared with the open group. Our meta-analysis revealed no statistically significant difference in hallux valgus angle (HVA), first intermetatarsal angle (IMA), the first metatarsal shortening, the American Orthopedic Foot and Ankle Society (AOFAS) score, visual analog scale (VAS) score at the final follow-up or complication rate (when all studies were considered). When taking into consideration only randomized controlled trial (RCT), the AOFAS score was higher in the MIS group while HVA, IMA, DMAA, and complication rate remained no significance. Post-operative IMA of the MIS group was significantly lower when only studies reporting the second-generation (2G) MIS were included. When just studies adopting the third-generation (3G) MIS were included, the HVA and DMAA were lower in the MIS group.

**Conclusion:**

The MIS procedures were more effective than open surgeries in the treatment of hallux valgus. Moreover, the MIS group achieved better radiologic and clinical outcomes compared with the open group.

## Introduction

Hallux valgus is a common forefoot disorder involving a lateral deviation of the hallux and medial deviation of the first metatarsal (1). Hallux valgus is often associated with first metatarsophalangeal joint osteoarthritis, and it has been linked to notable health problems, such as disability, a greater risk of falling, impaired balance, and lesser quality of daily life (2). Symptomatic hallux valgus is usually treated with surgeries such as chevron osteotomy, Lapidus procedure, and scarf osteotomy (3–7). There are over 150 open surgical procedures for hallux valgus, but none of them have been proven to be better than others. In recent years, minimally invasive surgery (MIS) has become popular for hallux valgus because of its shorter operation time, smaller scar, and shorter recovery time compared with open surgeries. So far, three generations of minimally invasive techniques have been developed. The first generation was the Reverdin–Isham technique (8), which used angular medial closed wedge osteotomy without osteosynthesis. The second generation (2G) was the Bösch osteotomy (9), which was a modification of Hohmann osteotomy. Temporary internal fixation with Kirschner wires were used after distal transverse osteotomy. The third generation (3G) was minimally invasive chevron and akin (MICA) (10), which used percutaneous osteotomies and was fixed with compression screws.

Recently, two meta-analyses (11, 12) have been carried out to determine the effects of MIS vs. open surgery, showing no significant difference in radiological outcomes and functional scores. However, relatively a few outcome measures (OMs) had been included in Singh's meta-analysis (11). Lu et al. conducted a meta-analysis in which most of the included studies were of low or moderate quality (12). In the last 3 years, several new studies (13–18) performed a comparison between MIS and open surgery for hallux valgus have reported. Therefore, we conducted this updated meta-analysis and included more OMs to comprehensively evaluate the efficacy of MIS vs. open surgery for hallux valgus.

## Materials and Methods

This meta-analysis was conducted in accordance with the preferred reporting items for systematic reviews and meta-analyses (PRISMA) statement (19) and the Cochrane Handbook guidelines (20).

### Search Strategy

A systematic literature search of Pubmed, Embase, and the Cochrane Library was performed from January 1, 1980 to October 1, 2021, using the following item: (“Hallux Abductovalgus” OR “Hallux valgus” OR “Bunion”) AND (“Percutaneous” OR “Bosch” OR “minimally invasive surgery” OR “minimally invasive” OR “Bösch” OR “SERI” OR “simple, effective, rapid, inexpensive” OR “minimally invasive chevron-Akin” OR “percutaneous chevron-Akin”), without a language filter.

### Inclusion and Exclusion Criteria

All studies included in this meta-analysis need to meet the following criteria: (1) comparative studies reporting comparisons of MIS vs. open surgery for hallux valgus; (2) patients with hallux valgus; (3) at least a 6-month follow-up; and (4) OMs including at least one of the following: hallux valgus angle (HVA), first intermetatarsal angle (IMA), the American Orthopedic Foot and Ankle Society (AOFAS) score, visual analog scale (VAS) score, operating time, and complications. The exclusion criteria were: (1) unpublished data; (2) case series, case reports, reviews, and proceedings of meetings; (3) biomechanical research; and (4) no available data describing OMs mentioned earlier.

### Data Extraction and Quality Assessment

Duplicates were initially excluded using Endnote Version X9. Two investigators independently screened titles and abstracts of the remaining studies. Then, full texts of the remaining studies were reviewed for eligibility according to the inclusion and exclusion criteria. Two independent reviewers performed data extraction from the included studies. The following data were extracted: the time of publication, country, study design, sample size, patients' age, technique, the duration of follow-up, HVA, IMA, AOFAS, and complications. Two authors used the Cochrane Handbook for Systematic Reviews of Interventions 5.2.0. (20) to assess the methodological quality and risk of bias of randomized controlled trials (RCTs), while the methodological qualities and risk of bias of non-RCTs were evaluated by the methodological index for non-randomized studies (MINORS) (21). MINORS score > 14 was set as the level of inclusionw. Disagreements were resolved by a discussion to reach a consensus.

### Statistical Analysis

The data analysis was performed with Review Manager (Version 5.3; The Cochrane Collaboration, Oxford, UK) and the statistical software R 4.0.3 with the meta package. For dichotomous data, the risk ratio (RR) along with 95% confidence intervals (CIs) was calculated. For continuous data, standardized mean difference (SMD) with 95% CIs was calculated. *p* < 0.05 indicated a statistical significance. *I*^2^-test was used to evaluate the heterogeneity between studies. When *I*^2^ > 50% and *p* < 0.10, the heterogeneity was significant, and a random effect model was used. Otherwise, a fixed-effects model was applied in the meta-analysis. Subgroup meta-analysis was conducted according to study design (RCT or non-RCT) and technique (2G or 3G MIS). The publication bias was assessed by the Egger' test.

## Results

### Characteristics of the Included Studies

**Figure 1:**
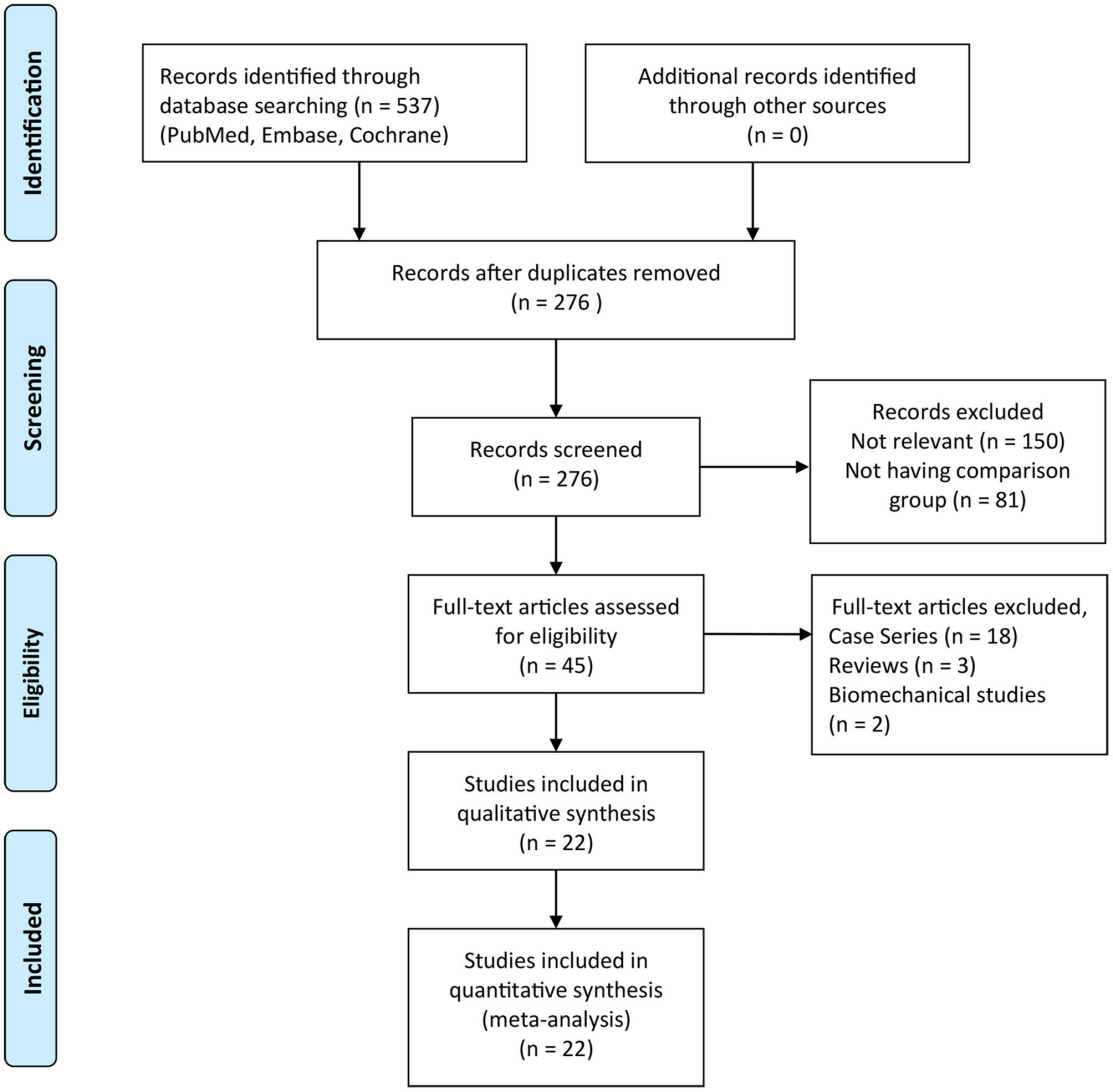
Flow diagram of the study selection process.

**Figure 2:**
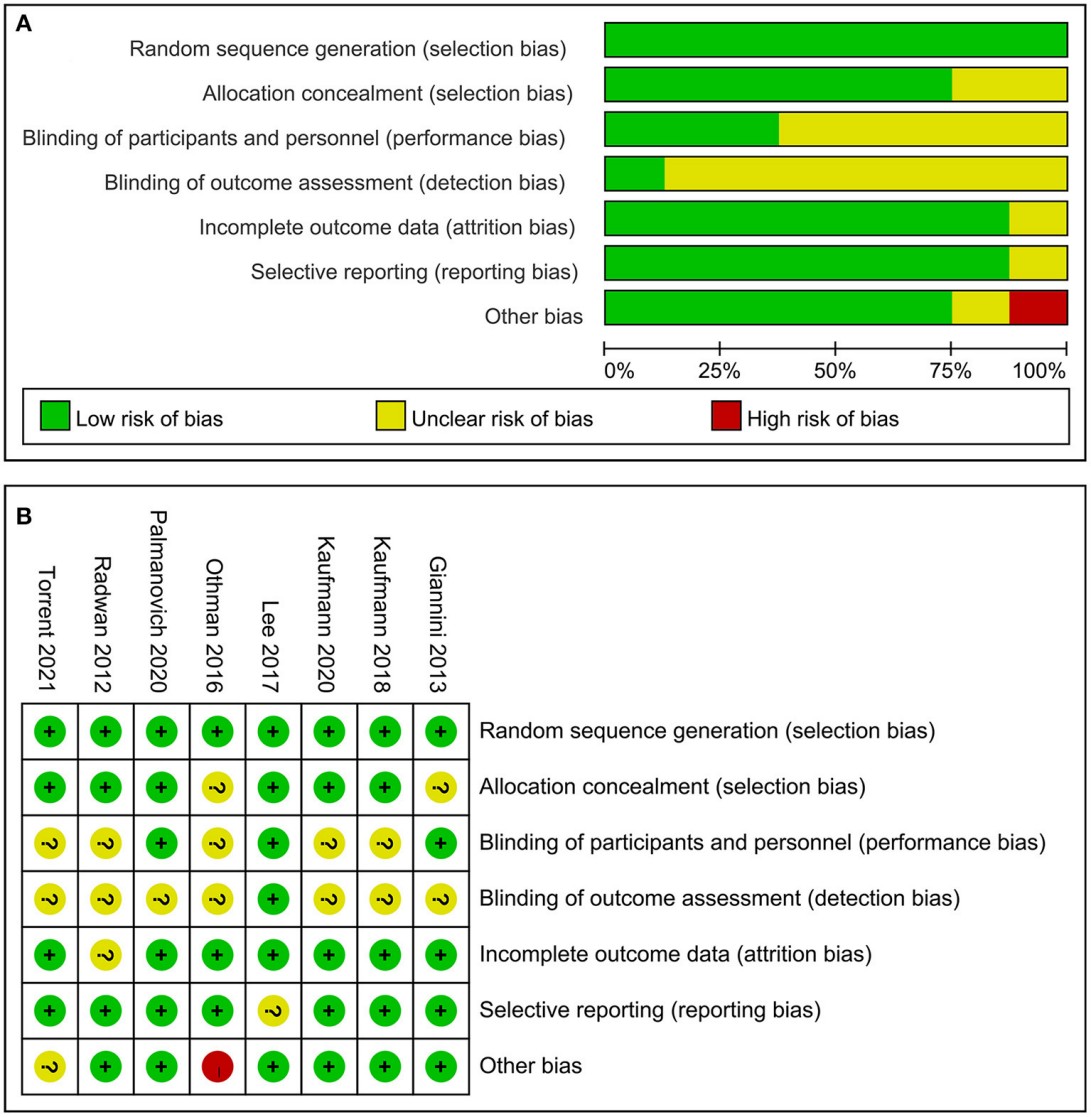
Quality assessment for randomized controlled trials (RCTs). **(A)** Risk of bias summary and **(B)** risk of bias graph.

As presented in Figure 1, a total of 537 articles were identified after the primary literature search. After the removal of duplicates, 276 articles remained. During the screening of titles and abstracts of the remaining studies, 150 irrelevant articles and 81 articles without a comparison group were excluded, and a total of 45 articles remained. Then, full-text reviewing according to inclusion and exclusion criteria was conducted. A total of 23 articles (including 18 case series, 3 reviews, and 2 biomechanical studies) were eliminated. Eventually, 22 studies were included in this meta-analysis, in which 790 ft were treated with MIS procedure and 838 ft with the open procedure. The characteristics of the included studies are depicted in Table 1. Follow-up times were highly variable, ranging from 6 months to 7 years. Among the included studies, 8 studies (15, 17, 24, 26, 28, 29, 31, 35) were RCTs, 2 studies (4, 14) were prospective comparative studies, and 12 studies (5, 13, 16, 18, 22, 23, 25, 27, 30, 32–34) were retrospective comparative studies. A total of 10 studies (13, 17, 22–28, 32) reported on the 2G percutaneous hallux valgus surgery, in which 7 studies (13, 22–25, 28) involved Bösch osteotomy and 3 studies (17, 26, 27) involved a simple, an effective, a rapid, and an inexpensive (SERI) technique. Roth et al. (22) conducted a retrospective comparative study to compare between Bösch and Kramer osteotomies. Maffulli et al. (23) performed a comparison between the Bösch technique and open Scarf osteotomy. Radwan and Mansour (24) performed a RCT to compare between Bösch and Chevron osteotomies. Chiang et al. (25) performed a retrospective comparison between Bösch and Ludloff osteotomies. Giannini et al. (26) conducted a RCT to determine the efficiency of the SERI technique and scarf surgery. Poggio et al. (27) conducted a retrospective study to compare between the SERI technique and open scarf technique for hallux valgus. Othman and Hegazy (28) performed a RCT to compare between Bösch surgery and open Chevron osteotomy. Choi et al. (13) performed a retrospective comparison between the Bösch technique and Chevron surgery. Palmanovich et al. (17) conducted a RCT to compare between the SERI and Chevron technique. Siddiqui et al. (32) performed a retrospective comparison between Bösch and Chevron surgery. Nine studies (5, 14–16, 18, 29, 30, 34, 35) reported on the 3G percutaneous hallux valgus surgery, including percutaneous chevron–akin (PECA) and MICA. Brogan et al. (5) performed a retrospective study to compare between the PECA and Chevron technique. Lee et al. (29) conducted a RCT to compare between the PECA and open scarf/akin technique. Lai et al. (30) performed a retrospective comparison between the PECA and scarf/akin technique. Kaufmann et al. (35) conducted a RCT to compare between the MICA and chevron/akin technique. Frigg et al. (14) performed a prospective comparative study to compare between the MICA and scarf/akin technique. Lim et al. (16) performed a retrospective comparison between MICA and scarf surgery. Schilde et al. (18) conducted a retrospective comparative study to compare between the MICA and scarf/akin technique. Tay et al. (34) performed a retrospective study to compare between the MICA and scarf/akin surgery. Two studies (4, 31) performed a comparison of the mini-scarf with open scarf osteotomy. Guo et al. (33) conducted a retrospective comparative study for a comparison of percutaneous oblique osteotomy (POO) with open chevron osteotomy. The methodological quality of RCTs is shown in Figure 2. The MINORS score of comparative studies was 17.4 ± 2.0 (ranged from 15 to 21) (Table 2), which indicated a high quality of the included studies.

**Table 1:** Characteristics of the included studies.

**References**	**Country**	**Design**	**No. of feet**		**Age (years)**		**Technique used**		**Follow-up (months)**	
			**MIS**	**Open**	**MIS**	**Open**	**MIS**	**Open**	**MIS**	**Open**
Roth et al. (22)	Austria	Retrospective	88	36	49	50	Bosch	Kramer	17	17
Maffulli et al. (23)	UK	Retrospective	36	36	51.5	52.6	Bosch	Scarf	25	25
Radwan and Mansour (24)	Egypt	RCT	29	31	32.7	35.7	Bosch	Chevron	21.7	19.5
Chiang et al. (25)	China Taiwan	Retrospective	32	30	61.1	64.5	Bosch	Ludloff	24	24
Giannini et al. (26)	Italy	RCT	20	20	53	53	SERI	Scarf	84	84
Poggio et al. (27)	Spain	Retrospective	69	133	62.5	52.9	SERI	Scarf	12	12
Brogan et al. (5)	UK	Retrospective	49	32	53	57	PECA	Chevron	31	37
Othman and Hegazy (28)	Egypt	RCT	33	25	40.47	39.2	Bosch	Chevron	49.36	51.56
Lee et al. (29)	Australia	RCT	25	25	52.6	53.4	PECA	Scarf/Akin	6	6
Lai et al. (30)	Singapore	Retrospective	29	58	54.3	54.3	PECA	Scarf/Akin	24	24
Kaufmann et al. (15)	Austria	RCT	25	22	52	44	MICA	Chevron/Akin	9	9
Boksh et al. (4)	UK	Prospective	16	21	52.2	46	Mini-Scarf	Scarf	28	28
Choi et al. (13)	South Korea	Retrospective	25	30	21.3	22.4	Bosch	Chevron	19.9	20.5
Frigg et al. (14)	Switzerland	Prospective	48	50	48.04	48.23	MICA	Scarf/Akin	24	24
Palmanovich et al. (17)	Israel	RCT	20	15	38.7	49.2	SERI	Chevron	38.7	49.2
Lim et al. (16)	Singapore	Retrospective	52	52	48.7	52.3	MICA	Scarf	48.7	52.3
Kaufmann et al. (15)	Austria	RCT	19	20	54	47	MICA	Chevron/Akin	54	47
Schilde et al. (18)	Germany	Retrospective	124	86	56.8	57.1	MICA	Scarf/Akin	56.8	57.1
Torrent et al. (31)	Spain	RCT	30	28	60.7	64.2	Mini-Scarf	Scarf	21	21
Siddiqui et al. (32)	USA	Retrospective	31	30	43.2	50	Bosch	Chevron	18.7	26.6
Guo et al. (33)	China	Retrospective	48	64	60.9	60.6	POO	Chevron	24	24
Tay et al. (34)	Singapore	Retrospective	30	30	51.7	52.7	MICA	Scarf/Akin	24	24

*RCT, Randomized controlled trial; NO., Number; MIS, Minimally invasive surgery; SERI, Simple, Effective, Rapid, Inexpensive; PECA, Percutaneous Chevron-Akin; MICA, Minimally invasive Chevron-Akin; POO, percutaneous oblique osteotomy*.

**Table 2:** MINORS score for each study to assess methodological quality.

**References**	**Methodological items**												**Total**
	**1**	**2**	**3**	**4**	**5**	**6**	**7**	**8**	**9**	**10**	**11**	**12**	
Roth et al. (22)	2	0	1	2	0	2	2	0	2	2	1	1	15
Maffulli et al. (23)	2	1	1	1	1	2	1	0	2	2	1	2	16
Chiang et al. (25)	2	1	2	2	1	2	2	0	2	2	1	2	19
Poggio et al. (27)	2	2	2	2	0	2	1	0	2	2	2	2	19
Brogan et al. (5)	2	2	2	2	0	2	0	2	2	2	2	1	19
Lai et al. (30)	2	0	2	2	1	2	2	2	2	2	2	2	21
Boksh et al. (4)	2	2	2	1	1	2	2	0	1	2	2	1	18
Choi et al. (13)	2	1	2	2	0	1	0	0	1	2	2	2	15
Frigg et al. (14)	2	2	2	1	2	2	2	0	1	2	2	2	20
Schilde et al. (18)	2	1	1	1	1	1	1	0	1	2	2	2	15
Lim et al. (16)	2	0	1	2	0	1	2	0	2	2	2	2	16
Siddiqui et al. (32)	2	1	1	1	1	2	0	0	2	2	1	2	15
Guo et al. (33)	2	2	2	1	1	1	2	0	1	2	2	1	17
Tay et al. (34)	2	2	2	1	1	2	2	0	1	2	2	1	18

*The final score comprises the results of 8 items or 12 items in cases of comparative studies: 1 A clearly stated aim; 2 Inclusion of consecutive patients; 3 Prospective collection of data; 4 Endpoints appropriate to the aim of the study; 5 Unbiased evaluation of the study endpoint; 6 Follow-up period appropriate to the aim of the study; 7 Loss to follow-up <5%; 8 Prospective calculation of the study size; 9 An adequate control group; 10 Contemporary groups; 11 Baseline equivalence of groups; 12 Adequate statistical analysis*.

### Radiologic Outcomes

#### Hallux Valgus Angle

**Figure 3:**
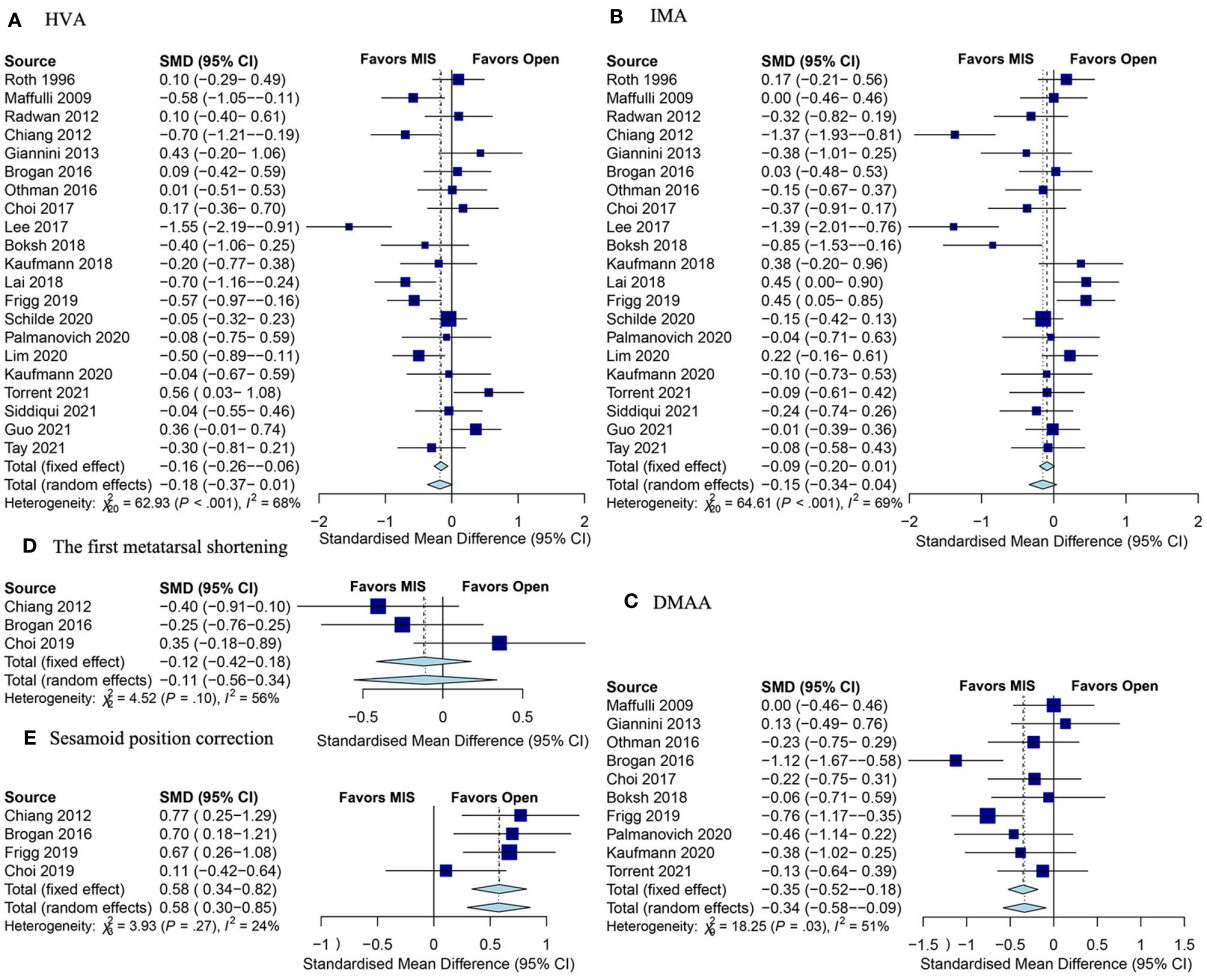
Forest plots of radiologic outcomes. **(A)** HVA between the MIS and open groups; **(B)** IMA; **(C)** DMAA; **(D)** the first metatarsal shortening; and **(E)** the medial sesamoid position correction. HVA, hallux valgus angle; MIS, minimally invasive surgery; IMA, first intermetatarsal angle; DMAA, distal metatarsal articular angle.

A total of 21 studies reported post-operative HVA. When evaluating all studies, the pooled SMD of HVA at the final follow-up was not significant between MIS and open groups (Figure 3A). When only studies reporting 3G MIS were included, the post-operative HVA was significantly lower in the MIS group (SMD: −0.4; 95% CI −0.68–0.13; *p* = 0.004; *I*^2^ = 69%;), but this significance was lost when comparing the just studies reporting 2G MIS (Table 3). Eight RCTs evaluated HVA, which did not reach a statistical significance (SMD: −0.08; 95% CI −0.50–0.34; *p* = 0.53; *I*^2^ = 76%; Table 3).

**Table 3:** Main results of subgroup meta-analysis.

**Index**	***n***	**RR**	**SMD**	**95% CI**	***P***	***I*^2^ %**	***P* for heterogeneity**	**Model**
HVA (RCT only)	8	NA	−0.08	−0.50 ~ 0.34	0.53	76	*P* < 0.01	REM
HVA (non-RCT only)	13	NA	−0.2	−0.43 ~ 0.03	0.03	63	*P* < 0.01	REM
HVA (2G MIS)	9	NA	−0.08	−0.31 ~ 0.15	0.51	46	0.06	FEM
HVA (3G MIS)	9	NA	−0.4	−0.68 ~ 0.13	*P* < 0.01	69	*P* < 0.01	REM
IMA (RCT only)	9	NA	−0.25	−0.58 ~ 0.08	0.14	63	*P* < 0.01	REM
IMA (non-RCT only)	13	NA	−0.09	−0.33 ~ 0.14	0.42	72	*P* < 0.01	REM
IMA (2G MIS)	9	NA	−0.28	−0.57 ~ 0.01	0.04	64	*P* < 0.01	REM
IMA (3G MIS)	9	NA	0.01	−0.30 ~ 0.31	0.96	75	*P* < 0.01	REM
DMAA (RCT only)	5	NA	−0.19	−0.46 ~ 0.06	0.14	0	0.72	FEM
DMAA (non-RCT only)	5	NA	−0.44	−0.87 ~ 0.02	0.04	71	*P* < 0.01	REM
DMAA (2G MIS)	5	NA	−0.14	−0.38 ~ 0.11	0.27	0	0.71	FEM
DMAA (3G MIS)	3	NA	−0.79	−1.08 ~−0.49	*P* < 0.01	35	0.21	FEM
AOFAS score (RCT only)	7	NA	0.45	0.03 ~ 0.87	0.04	73	*P* < 0.01	REM
AOFAS score (non-RCT only)	10	NA	0.14	−0.25 ~ 0.53	0.48	89	*P* < 0.01	REM
AOFAS score (2G MIS)	7	NA	0.37	−0.17 ~ 0.90	0.18	88	*P* < 0.01	REM
AOFAS score (3G MIS)	8	NA	0.17	−0.22 ~ 0.56	0.39	83	*P* < 0.01	REM
Complication rate (RCT only)	8	1.03	NA	0.68 ~ 1.57	0.89	28	0.21	FEM
Complication rate (non-RCT only)	12	1.12	NA	0.70 ~ 1.82	0.62	52	0.01	REM
Complication rate (2G MIS)	9	1.05	NA	0.55 ~ 2.02	0.88	65	*P* < 0.01	REM
Complication rate (3G MIS)	9	1.07	NA	0.76 ~ 1.51	0.71	0	0.47	FEM

*N, number of included studies; RR, risk ratio; SMD, standardized mean difference; CI, confidence interval; RCT, randomized controlled trial; HVA, hallux valgus angle; 2G, second generation; 3G, third generation; MIS, minimally invasive surgery; NA, not applicable; FEM, fixed effect model; REM, random effect model; IMA, first intermetatarsal angle; DMAA, distal metatarsal articular angle; AOFAS, the American Orthopedic Foot and Ankle Society*.

#### First Intermetatarsal Angle

First intermetatarsal angle was assessed in 21 studies, and there was no significant difference in the post-operative IMA between these two groups (Figure 3B). Nine RCTs documented IMA post-operatively, which demonstrated no statistical significance between MIS and open groups (SMD: −0.25; 95% CI −0.58–0.08; *p* = 0.14; *I*^2^ = 63%; Table 3). 2G MIS was assessed in 9 studies, which found that IMA was significantly lower in the MIS group (SMD: −0.28; 95% CI −0.57–0.01; *p* = 0.04; *I*^2^ = 64%; Table 3). However, the significance lost when comparing the studies reporting 3G MIS (SMD: 0.01; 95% CI −0.30–0.31; *p* = 0.96; *I*^2^ = 75%; Table 3).

#### Distal Metatarsal Articular Angle

A total of 10 studies provided a post-operative distal metatarsal articular angle (DMAA). The post-operative DMAA was significantly lower in the MIS group compared with the open group (SMD: −0.34; 95% CI −0.58–0.08; *Z* = −2.67; *p* = 0.007; *I*^2^ = 51%; Figure 3C). Three studies reported DMAA after 3G MIS, in which the post-operative DMAA was significantly lower in the MIS group (SMD: −0.79; 95% CI −1.08 to −0.49; *p* < 0.01; *I*^2^ = 35%; Table 3). Nevertheless, there was no significance between the MIS and open group when just comparing the 3G MIS studies (SMD: −0.14; 95% CI −0.38–0.11; *p* = 0.27; *I*^2^ = 0%; Table 3).

#### The First Metatarsal Shortening

Three studies assessed the first metatarsal shortening, in which there were 98 ft treated with the MIS procedure and 84 ft treated with the open procedure. As shown in Figure 3D, the pooled SMD was also not significant between these two groups.

#### Sesamoid Position Correction

A total of 4 studies (5, 13, 14, 25) reported the medial sesamoid position as demonstrated by Hardy and Clapham (36). The pooled results showed that there were more sesamoid position changes in the MIS group compared with the open group (SMD: 0.58; 95% CI 0.34–0.82; *Z* = 4.69; *p* < 0.001; *I*^2^ = 24%; Figure 3E).

### Clinical Outcomes

#### AOFAS Score

**Figure 4:**
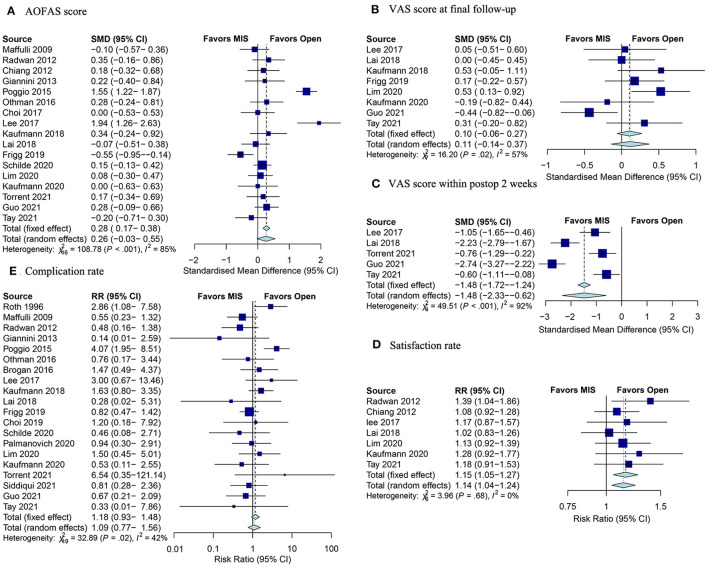
Forest plots of clinical outcomes. **(A)** SMD for the AOFAS score improvement between the MIS and open groups; **(B)** VAS score at the final follow-up; **(C)** VAS score within post-operative 2 weeks; **(D)** satisfaction rate; and **(E)** complication rate. SMD, standardized mean difference; AOFAS, the American Orthopedic Foot and Ankle Society; MIS, minimally invasive surgery; VAS, visual analog scale; postop, post-operative.

The AOFAS score (37) was available in 17 studies, and no significant difference between MIS and surgery groups was observed (Figure 4A). Seven RCTs reported the AOFAS score, and demonstrated a higher score in the MIS group (SMD: 0.45; 95% CI 0.03–0.87; *p* = 0.04; *I*^2^ = 73%; Table 3). Subgroup meta-analysis of techniques (2G or 3G MIS) found no significant difference (Table 3).

#### VAS Score

Eight studies were incorporated in the analysis of VAS score at the final follow-up (38). The overall results indicated no significant difference between MIS and open groups (Figure 4B). Besides, 5 studies assessed the VAS score within post-operative 2 weeks. The pooled results showed that the MIS procedure was associated with obviously less pain in the early post-operative phase (SMD: −1.48; 95% CI −2.33–0.62; *Z* = −3.38; *p* < 0.001; *I*^2^ = 92%; Figure 4C).

#### Satisfaction Rate

A total of 7 studies documented the satisfaction rate. The pooled results indicated that the satisfaction rate was remarkably higher in the MIS group (RR: 1.15; 95% CI: 1.05–1.27; *Z* = 3.09; *p* = 0.002; *I*^2^ = 0%; Figure 4D).

#### Complication Rate

There were some post-operative complications in MIS and open surgery for hallux valgus, such as screw irritation, recurrence, and non-union. In total, 20 studies reported complication rates. According to the pooled results, there was no difference between MIS and open groups with respect to the complication rate (Figure 4E). Subgroup analysis of study design (RCT or non-RCT) and techniques (2G or 3G MIS) demonstrated no significance difference in the complication rate between the two groups (Table 3).

### Secondary Outcomes

#### Duration of Surgery

**Figure 5:**
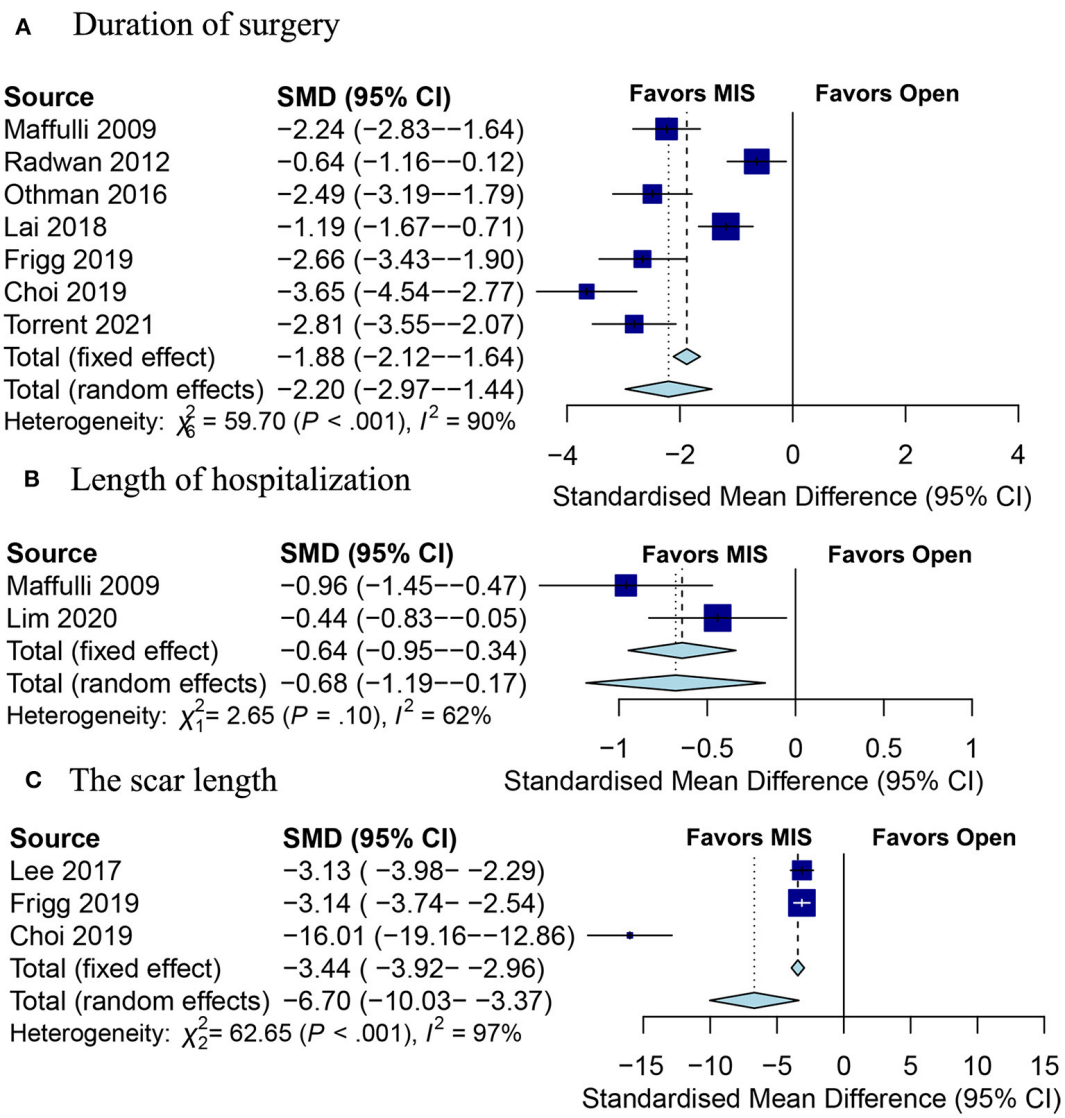
Forests plot of secondary outcomes between the MIS and open groups. **(A)** Duration of surgery; **(B)** length of hospitalization; **(C)** the scar length. MIS, minimally invasive surgery.

The duration of surgery was available in 7 studies. Figure 5A showed that the pooled SMD was statistically significant (SMD: −2.81; 95% CI −3.55 to −2.07; *Z* = −5.64; *p* < 0.001; *I*^2^ = 90%), indicating the less duration of surgery in the MIS group in comparison with the open group.

#### Length of Hospitalization

Two studies (16, 23) assessed the length of hospitalization, and the pooled results demonstrated that the MIS group was associated with a shorter length of hospitalization (SMD: −0.64; 95% CI −0.95 to −0.34; *Z* = −4.13; *p* < 0.001; *I*^2^ = 62%; Figure 5B).

#### The Scar Length

Three studies (13, 14, 29) documented the scar length. The pooled results indicated that the scar length was less in the MIS group as compared to the open group (SMD: −6.70; 95% CI −10.03 to −3.37; *Z* = −3.94; *p* < 0.001; *I*^2^ = 97%; Figure 5C).

### Publication Bias

The Egger' test of HVA suggested that there was no obvious publication bias (*p* = 0.58).

## Discussion

More than 150 open surgical procedures have been described for the treatment of hallux valgus (39). There was a trend toward more minimally invasive surgery for hallux valgus, involving the theoretical advantages of less soft tissue dissection, reduced surgical time, and a faster recovery time (3). In this meta-analysis, MIS procedure showed better radiologic and clinical outcomes compared with the open group.

Compared to the former meta-analysis conducted by Singh et al. (11), 13 new studies were included in this meta-analysis, making it more comprehensive. Most of the included studies in the meta-analysis performed by Lu et al. (12) were of low or moderate quality, and limited OMs were assessed due to incomplete information. Our meta-analysis included all new studies according to the inclusion and exclusion criteria and pooled 12 OMs.

Hallux valgus angle and IMA were the most common radiologic OMs to evaluate hallux valgus because they have been shown to determine the severity of hallux valgus. IMA correction was a marker to determine the corrective potential of a metatarsal osteotomy (40). Singh et al. (11) pooled 8 studies for HVA and IMA, showing no significant difference between MIS and open groups. Our meta-analysis found no significant difference in HVA and IMA between the two groups when all 22 studies were included. Nevertheless, post-operative IMA of the MIS group was significantly lower when only studies reporting 2G MIS were included. The 2G MIS was Bösch osteotomy or the SERI technique, which adopted an axial wire to displace and maintain the metatarsal head in the initial few weeks (5). When just studies adopted 3G MIS were included, the HVA were lower in the MIS group. Lai et al. (30) performed a comparison between the PECA and open scarf/akin technique, showing a better HVA correction but a comparable IMA correction in the MIS group. The 3G MIS included MICA and PECA. PECA is a modification from the MICA procedure as introduced by Vernois and Redfern (10). The MICA technique adopted phalangeal osteotomy with burr, which had a direct impact on the correction of HVA (35). Lim et al. (16) also demonstrated lower post-operative HVA in the MIS group, and they believed that it was possibly owing to the use of toe alignment splint after the MICA procedure. DMAA is the angle between the perpendicular line to the distal metatarsal articular surface and the first metatarsal axis. A pathological joint line is associated with a significantly increased recurrence rate (41), so it is essential to restore the DMAA. Brogan et al. (5) reported a trend toward a difference in DMAA in favor of the PECA group compared with the open chevron group, perhaps because of the effect of the wire causing a slight varus displacement of the metatarsal head fragment. Frigg et al. (14) performed a retrospective comparison of MICA with open scarf-akin surgery for hallux valgus and reported a lower DMAA in the MICA group. This meta-analysis pooled the data from 10 studies reporting DMAA, indicating a lower DMAA in the MIS group.

The diameter of burrs used in MIS procedures is about 2 mm, which may lead to shorter metatarsal compared with open techniques (5). The shortened first metatarsal will transfer more load to the lateral metatarsal heads, which could cause metatarsalgia (42). This meta-analysis indicated that MIS surgery do not cause more metatarsal shortening compared with open surgery.

The change of the sesamoid position was better in the MIS group than in the open group, which perhaps was due to more use of image intensifier. Okuda et al. (43) pointed out that insufficient sesamoid position correction was an important risk factor for hallux valgus recurrence, so the correction of the sesamoid position was needed.

The AOFAS score (37) was to evaluate the functional outcome, including pain (40 points), function (45 points), and alignment (15 points). Singh et al. (11) conducted a meta-analysis to demonstrate that the open group provided more improvement in the AOFAS score. Poggio et al. (27) reported that the open scarf technique showed more increases in the AOFAS score than the Kramer technique (also known as the 2G MIS technique). Radwan and Mansour (24) and Lee et al. (29) reported a trend toward a better improvement in the AOFAS score in the MIS group. This meta-analysis pooled 17 studies and found no difference in the improvement of the AOFAS score between the two groups. When taking into consideration only RCTs, the AOFAS score was higher in the MIS group. The VAS score of the early post-operative phase was lower in the MIS group, which reflected that the MIS surgery required a minor soft tissue dissection (15). The VAS score at the final follow-up was not found to be significant, which was in accordance with Singh's meta-analysis.

Singh's meta-analysis pooled 4 studies and found no significant difference in the satisfaction rate between MIS and open groups. Lu's meta-analysis pooled 3 studies and demonstrated a higher satisfaction level in the MIS group. Our meta-analysis included 7 studies, showing a higher satisfaction rate in the MIS group. The high satisfaction rate in the MIS was possibly due to the cosmetic result of surgery. Choi et al. reported 2G MIS for young female patients, and believed that the MIS technique could be considered for young female patients who desire a less visible scar (13). The scar length was significantly shorter in the MIS group. Because of the limited scar on the medial side, MIS is expected to result in fewer soft tissue complications, less stiffness, and a higher satisfaction rate.

The duration of surgery and the length of hospitalization were shorter in the MIS group, possibly due to the limited exposure and steps involved in the MIS technique (23). The shorter length of hospitalization in the MIS group made the MIS procedure a beneficial choice for high-risk patients suffering recurrent ulceration (23). However, Lai et al. reported that the fluoroscopy time was longer in the MIS group compared with the open group, involving higher radiation exposures (30).

Jowett et al. described a steep learning curve for MIS techniques. Jowett et al. made a comparison of a single surgeon series and found a higher reoperation rate and a lower satisfaction rate in the first 53 ft compared with the subsequent 53 ft (44). To minimize the learning curve, cadaveric training was recommended for any surgeon considering performing MIS surgery (30).

To our knowledge, this meta-analysis includes the largest number of studies (22 studies) evaluating the efficiency of MIS vs. open surgery for hallux valgus. There were some limitations in this meta-analysis. First, different surgical techniques were used in the MIS and open groups, involving a high heterogeneity. Secondly, non-randomized controlled studies were included; therefore, the results of this study must be interpreted with caution due to the natural defects of retrospective studies. Larger sample multicenter randomized controlled studies are needed to further verify the results of this meta-analysis.

## Conclusion

The MIS procedures were more effective than open surgery in the treatment of hallux valgus. Moreover, the MIS group achieved better radiologic and clinical outcomes compared with the open group. The 2G MIS demonstrated better corrective power to IMA while the 3G MIS provided a stronger correction to HVA. The MIS procedures offered benefits mainly in the early post-operative period, including a shorter surgery time, a more cosmetic scar, a higher satisfaction rate, and a faster recovery time. These features of the MIS procedure make it a better choice for young female patients who favor a cosmetic scar and patients who are at high risk due to recurrent ulceration.

## Data Availability Statement

The original contributions presented in the study are included in the article/supplementary material, further inquiries can be directed to the corresponding author/s.

## Author Contributions

MZ designed this study. KW and SD conducted the literature search and data extraction. SD and CS performed quality assessment of the included studies. LJ and SS performed a statistical analysis. LJ and KW wrote this manuscript. All authors have read and approved this final manuscript.

## Funding

This work was supported by the Natural Science Foundation of Beijing (Grant No. 7212020), Science and Technology Planning Project of Beijing Municipal Education Commission (Grant No. KM202110025013), and the Beijing Thousand Talents Project (Grant No. 2020A43).

## Conflict of Interest

The authors declare that the research was conducted in the absence of any commercial or financial relationships that could be construed as a potential conflict of interest.

## Publisher's Note

All claims expressed in this article are solely those of the authors and do not necessarily represent those of their affiliated organizations, or those of the publisher, the editors and the reviewers. Any product that may be evaluated in this article, or claim that may be made by its manufacturer, is not guaranteed or endorsed by the publisher.
